# Supplementary material for: A Global Screen for Assembly State Changes of the Mitotic Proteome by SEC-SWATH-MS
Source: Cell Syst. 2020 Feb 26;10(2):133–155.e6. doi: 10.1016/j.cels.2020.01.001 (PMC7042714; doi:10.1016/j.cels.2020.01.001)

Q9NRL2 | BAZ1A_HUMAN | BAZ1A ACF1 WCRF180 HSPC317
Monomer MW [kDa]: 178.702 Monomer expected elution fraction: 35

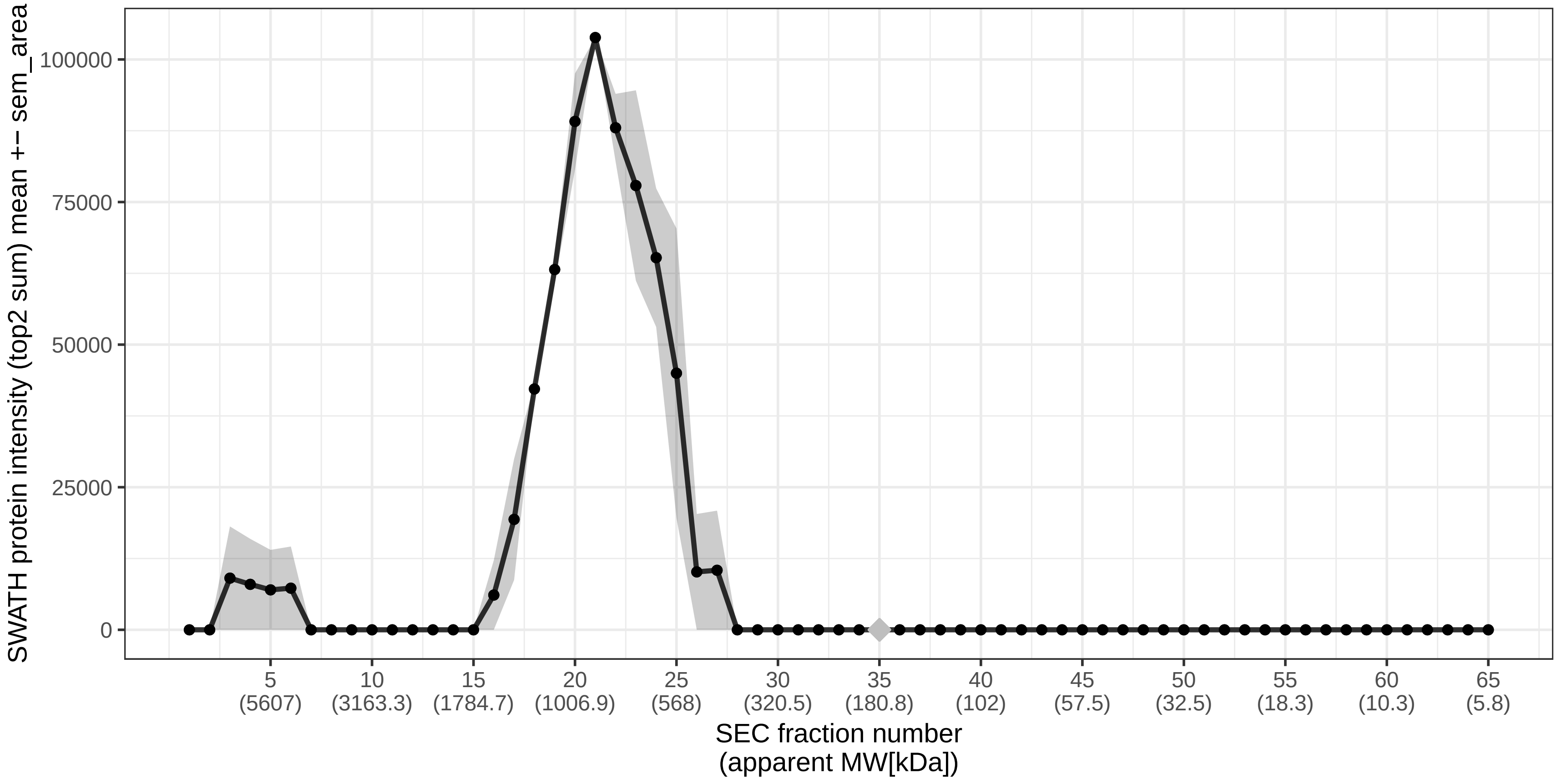

Supplement: Data S1. SEC-SWATH-MS Protein Chromatograms, Related to Figure 1 [file mmc6.zip › SECchrom_Q9NRL2_BAZ1A_HUMAN_BAZ1A_ACF1_WCRF180_HSPC317.pdf]
